# Awareness and outcomes of the fruits and veggies (FNV) campaign to promote fruit and vegetable consumption among targeted audiences in California and Virginia: a cross-sectional study

**DOI:** 10.1186/s12889-021-11055-6

**Published:** 2021-06-09

**Authors:** Tessa R. Englund, Valisa E. Hedrick, Sofía Rincón-Gallardo Patiño, Lauren E. Kennedy, Kathryn W. Hosig, Elena L. Serrano, Vivica I. Kraak

**Affiliations:** 1 Thurston Arthritis Research Center, University of North Carolina at Chapel Hill, 3300, Thurston Building, CB 7280, Chapel Hill, NC 27599 USA; 2 Department of Human Nutrition, Foods, and Exercise, Virginia Tech, 335-A Wallace Hall, 295 West Campus Drive, Blacksburg, Virginia, 24061 USA; 3 Department of Human Nutrition, Foods, and Exercise, Virginia Tech, 338 Wallace Hall, 295 West Campus Drive, Virginia Tech, Blacksburg, Virginia, 24061 USA; 4 Michigan State University Extension, Justin S. Morrill Hall of Agriculture, 446 W. Circle Drive, Room 409, East Lansing, MI 48824-1039 USA; 5 Department of Population Health Sciences, Virginia Tech, Virginia-Maryland College of Veterinary Medicine, Room 334, 205 Duck Pond Drive, Blacksburg, VA 24061 USA; 6 Department of Human Nutrition, Foods, and Exercise, Virginia Tech, 327 Wallace Hall, 295 West Campus Drive, Blacksburg, Virginia, 24061 USA; 7 Department of Human Nutrition, Foods, and Exercise, Virginia Tech, 257 Wallace Hall, 295 West Campus Drive, Blacksburg, Virginia, 24061 USA

**Keywords:** Fruits, Vegetables, Social marketing, Program evaluation, Health promotion, Health behavior, Awareness, Food preferences

## Abstract

**Background:**

In 2015, the Partnership for a Healthier America launched the branded Fruits & Veggies (FNV) Campaign to apply a unique industry-inspired marketing approach to promote fruit and vegetable sales and intake to moms and teens in two US pilot markets: Fresno, California and Hampton Roads, Virginia. The aims of this cross-sectional study were to: 1) assess brand awareness and fruit- and vegetable-related outcomes among FNV Campaign target audiences in the California and Virginia market locations; and 2) examine whether reported awareness of the FNV Campaign was associated with differences in fruit- and vegetable-related cognitive and behavioral outcomes.

**Methods:**

Data for this cross-sectional study were collected using an online survey administered to a non-probability convenience sample (*n* = 1604; February–July 2017) of youth aged 14–20 years (*n* = 744) and moms aged 21–36 years (*n* = 860) in the two pilot markets. Descriptive statistics were computed and outcomes compared between unaware and aware respondents, controlling for sociodemographic covariates. Multivariate analysis of covariance (MANCOVA) was conducted to assess whether fruit- and vegetable-related attitude, belief, and encouragement outcomes differed by FNV Campaign awareness; logistic regression was used to examine associations between FNV brand awareness and dichotomous variables (fruit- and vegetable-related behavioral intentions, trying new fruits and vegetables); and ANCOVA was used to assess associations with daily fruit and vegetable intake frequency.

**Results:**

Approximately 20% (*n* = 315/1604) of respondents reported awareness of the FNV Campaign. Youth that reported awareness of the FNV Campaign (*n* = 167, 22.4%) had higher intentions to buy (*p* = 0.003) and eat (*p* = 0.009) fruits and vegetables than unaware respondents. Mothers that reported awareness of the FNV Campaign (*n* = 148, 17.2%) reported greater encouragement for friends and family to eat fruits and vegetables (*p* = 0.013) and were approximately 1.5 times more likely to report trying a new fruit or vegetable (*p* = 0.04) than mothers unaware of the Campaign. Daily fruit and vegetable intake frequency did not differ by Campaign awareness.

**Conclusions:**

FNV Campaign awareness was associated with limited but positive short- and intermediate-term cognitive and behavioral outcomes among target audience respondents. These findings can inform future research to enhance understanding and improve the FNV Campaign as it is expanded to new markets nationwide.

**Supplementary Information:**

The online version contains supplementary material available at 10.1186/s12889-021-11055-6.

## Background

The health benefits of consuming recommended amounts of fruits and vegetables are well recognized and include reduced risk of chronic non-communicable diseases such as cardiovascular disease, stroke, and cancer; and premature mortality [1–3]. However, the majority of children, adolescents, and adults in the United States (US) do not meet the Dietary Guidelines for Americans 2015–2020 recommendations for fruit and vegetable intake (2 and 2.5 cup-equivalents, respectively, for an adult consuming 2000 cal per day) to promote health and prevent chronic disease [4]. Despite heightened awareness about the benefits of consuming fruits and vegetables to support health, only 12.2 and 9.3% of US adults met fruit and vegetable recommended targets, respectively; and young adults ages 18–30 years have the lowest prevalence of meeting the fruit (9.2%) and vegetable (6.7%) intake recommendations [5]. A 2017 survey of US high-school students aged 14–18 years found that 7.1% of respondents met fruit intake recommendations and just 2.0% met vegetable intake recommendations [6]. Research suggests that large-scale interventions, including mass media and marketing campaigns, have potential to increase population fruit and vegetable intake [7].

To improve the effectiveness of large-scale campaigns, some researchers have called for a shift towards approaches common in commercial marketing campaigns to encourage healthy food-related behaviors, using creative and emotional appeals. This shift also includes a move away from education-based approaches traditionally used to increase awareness and knowledge about recommended intake levels and benefits of fruit and vegetable consumption in public health campaigns [8–11]. For example, commercial food and beverage marketing communication messages often feature creative, humorous, and emotional appeals designed to attract consumers’ attention and elicit positive affective (e.g., feelings and emotions) and cognitive (e.g., attitudes and intentions) responses and associations with brands and products that interact with a consumer’s experience to influence dietary behaviors [12, 13]. In contrast, previous national fruit and vegetable health-promotion campaigns, such as the *5 a Day for Better Health Program,* used informational approaches to raise awareness about the importance of consuming recommended servings of fruits and vegetables daily and improve knowledge, attitudes, and self-efficacy to increase consumption [14]. *5 a Day* was based on health behavior theories (i.e., Health Belief Model, Social Cognitive Theory, and Stages-of-Change Model) and applied social marketing techniques to reach consumers through multiple media channels and settings (e.g., school campaigns, worksite programs) [8, 14]. In 2007, *5 a Day* was rebranded and replaced by the *Fruits and Veggies–More Matters* Campaign with a new logo and messaging focused on the emotional benefits of fruit and vegetable consumption, which primarily appealed to mothers’ values as role models and leaders in supporting healthy eating for their families [15].

In 2015, the Partnership for a Healthier America (PHA), a non-profit organization working to leverage private-sector partnerships to improve the US food environment [16], introduced a new campaign with a unique approach to promote fruits and vegetables based on commercial marketing strategies. The PHA launched the branded Fruits & Veggies (FNV) Campaign in two pilot market locations; Fresno, California (CA) and Hampton Roads, Virginia (VA), to promote sales and intake of fruits and vegetables among targeted teen and mom audiences [17, 18]. The FNV Campaign was designed to present fruits and vegetables as fun and cool by using “humor and the power of celebrity to voluntarily shift consumer behavior toward healthier dietary choices” [19], on the basis that youth are more likely to consume foods endorsed by celebrities [16, 18]. In addition to targeting youth, targeting parents is particularly important as they can encourage intake, increase availability and accessibility of fruits and vegetables in the home, and be important role models for healthy eating behaviors [20]. Mothers are of particular interest as they spend more time with children on average and thus have greater potential to influence food preferences and eating behavior [21].

It is difficult to assess the impact of large-scale marketing campaigns and there is scarce empirical evidence from formative, process, and outcome evaluations of national diet-related campaigns [22]. Rekhy and McConchie reviewed past studies examining large-scale US public health campaigns that used informational approaches to promote fruit and vegetable consumption reported improvements in short- to medium-term outcomes, including awareness, attitudes, and behavioral intentions but have not demonstrated meaningful and sustained improvements in long-term consumption patterns (i.e., 5 years or more) [8]. The FNV Campaign has been included as an *emerging* evidence-based approach that requires further evaluation in the Supplemental Nutrition Assistance Program-Education (SNAP-Ed) Toolkit, which lists the campaign as a social marketing and Policy, Systems and Environmental (PSE) intervention [19]. While the initial pilot market campaigns were not implemented formally through SNAP-Ed, the PHA aimed to expand the FNV Campaign through SNAP-Ed and other partnerships across the US to reach low-income audiences [23]. As the FNV Campaign used an industry-inspired marketing approach that was not designed around a traditional health behavior theory and promoted fruit and vegetable products broadly, it is important to build the evidence base to understand the potential of this novel strategy to positively influence target audiences’ fruit and vegetable consumption. Understanding the short- to medium-term cognitive and behavioral outcomes of target audiences in the pilot markets can inform the FNV Campaign refinement and expansion, while also providing preliminary data and context to future assessments of long-term outcomes to determine if consumption patterns have changed or maintained years to decades on [8]. This is particularly important as formative and process research outcomes and timeline of the FNV Campaign development and pilot implementation are not well documented [22, 24].

This study was conducted to address the evidence gap regarding the use of commercial marketing strategies to promote fruits and vegetables by evaluating outcomes of the FNV Campaign in the initial launch markets through an online survey. The aims of this cross-sectional study were to: 1) assess awareness and fruit- and vegetable-related outcomes among respondents from the target audiences of the FNV Campaign (i.e., moms and teens) in the Fresno, CA and Hampton Roads, VA market locations; and 2) examine whether reported awareness of the FNV Campaign was associated with differences in fruit- and vegetable-related intermediate-term outcomes for attitudes, beliefs, encouragement, intentions, and long-term behavioral outcomes related to purchases and intake among the target audiences in the two markets.

## Methods

### FNV campaign context

The creative advertising firm, Victor & Spoils, designed the FNV Campaign marketing strategy, which was “inspired by big consumer brands, whose tactics are relentless, compelling, catchy, and drive an emotional connection with their products” [17]. The FNV Campaign strategy used integrated marketing communications (IMC) to engage target audiences with creative and humorous content and build positive associations with the FNV brand and fruits and vegetables. Marketers use IMC to reach consumers through multiple channels with targeted and synergized marketing communications designed to maximize reach and impact on brand preferences and behaviors [25]. The advertisements used visually appealing graphics and pro bono celebrity endorsers throughout IMC promotions, including multi-media advertising (https://fnv.com/), public relations, and event appearances. The underlying rationale was that celebrity endorsers could positively influence fruit and vegetable attitudes and behaviors of consumers [17, 18].

The PHA aimed to pilot the FNV Campaign in two racially and ethnically diverse markets over the first year to inform future efforts [26, 27]. Due to a variety of factors, including exposure to less healthy food environments, fruit and vegetable intake among Non-Hispanic Black populations in the US is generally lower than for Non-Hispanic whites, and in some cases Hispanic populations [28–30]. Hispanic and Black youth are of particular relevance for healthy marketing interventions as research has shown that they are disproportionately targeted by creative marketing promotions for nutritionally poor, processed foods and beverages [31, 32]. Over half of the Fresno County, CA population is of Hispanic ethnicity (52%) [33], which is about three times higher than the national population. In contrast, the Hampton Roads, VA metropolitan area has more than double the proportion of Black or African American residents (31%) than the national population [34].

Local pilot FNV Campaign execution began in June 2015 and included traditional media promotions through television, radio, print, in-store, and billboard advertising [16]. Digital and social media advertising complemented the ongoing local pilot campaign execution and supported reach of a wider audience through earned media (e.g., media coverage generated from external parties outside of the PHA). One year after the 2015 launch, the PHA reported that the FNV Campaign had garnered over 650 million impressions through earned media and 350 million impressions through social media [35]; additional outcomes related to reach and engagement were reported for the FNV Campaign in the pilot markets, but not from independent, peer-reviewed studies [16, 35, 36].

### Study design

This study was part of an independent evaluation of the FNV Campaign conducted by the research team between September 2015 and December 2017. The Robert Wood Johnson Foundation (RWJF) awarded a grant to one of the authors (VK) to evaluate the FNV Campaign, which was carried out by the research team after the PHA had launched the FNV Campaign in the pilot markets. Due to resource limitations and a lack of baseline (pre-intervention) data, alternative designs that included comparison groups (e.g., alternative markets) or pre- post- tests were not feasible, so a cross-sectional study design was used to assess short to medium-term outcomes (e.g., awareness, behavioral determinants) in the pilot markets.

Data for this study were collected from a cross-sectional online survey conducted among the target audiences from the two pilot markets between February and July 2017. The study aims, eligibility criteria, and measures used in the survey were developed in conjunction with the RWJF with input from the PHA staff. The Virginia Tech Institutional Review Board approved the study protocol that involved human subjects in December 2016 (IRB #15–1110).

The evaluation approach used to assess associations between awareness of the FNV Campaign and fruit- and vegetable-related outcomes in this cross-sectional study was similar to that used for the *Fruits and Veggies–More Matters* Campaign [37]. The *Fruits and Veggies–More Matters* Campaign was implemented nationally to encourage adults to consume fruits and vegetables, so the survey sample was recruited from a national panel of US adults. As the FNV Campaign was aimed at encouraging fruit and vegetable purchases and consumption among mom and teen audiences in two CA and VA markets, the recruitment efforts and eligibility criteria corresponded to these target population criteria.

The survey was pilot-tested to ensure that the procedure from participants initiating the survey to completion and compensation went as planned, and to ask for participants’ input on survey clarity and ease of use. A small sample of mothers (*n* = 3) and teens (*n* = 2) who lived outside of the pilot markets were recruited to take the survey and provide any feedback on the clarity and process for initiating and completing the survey. The survey procedure was executed as planned and participants did not report any issues or suggestions to improve the survey, so the team then began recruitment efforts in the two pilot Fresno, CA and Hampton Roads, VA markets.

#### Recruitment

Participants were recruited by members of the research team to complete an online survey through a non-probability convenience sampling strategy that was intended to reach available and accessible members of the target audiences living in Fresno, CA and Hampton Roads, VA. Recruitment in the two markets included community outreach with assistance of local organizations (e.g., daycare and youth activity centers, faith-based and social support non-profits) involved with the target populations, and distribution of print and digital flyers to local organizations that described the study, eligibility criteria, and provided a link to access the survey. Organizations were asked to post and/or email recruitment flyers to potentially eligible contacts. The main organizations involved with recruitment in Fresno included Head Start, Fresno Parks and Recreation, and the non-profits Cultiva La Salud and Centro La Familia; and the main organizations involved with recruitment in Virginia included Head Start, Virginia Cooperative Extension, and the Five Points Community Farmers Market.

The eligibility criteria and convenience sampling strategy for this survey were identified in coordination with the PHA and the RWJF based on the target audience demographic characteristics of the FNV Campaign at the time. Teens and young adults aged 14–20 years, who were part of the teen target audience at the time of the FNV Campaign launch in 2015, (hereafter referred to as teens/young adults), and mothers aged 21–36 years who were residents of Fresno, CA or Hampton Roads, VA were eligible to participate. Eligible participants could take part in the study if they could access the survey and complete it in either English or Spanish. Participants were instructed to use the survey link that corresponded to their demographic criteria so that those ages 14–20 years could participate in the adolescent/young adult survey and mothers aged 21–36 years could choose the mom survey.

Participants recruited from Fresno, CA and Hampton Roads, VA were screened prior to taking the survey to ensure that their demographic characteristics and residence met the eligibility criteria, by asking respondents to indicate their age range and place of residence. Participants received information about the research prior to participating and provided implied informed consent or assent by beginning the survey. As there was minimal to no risk involved in participating in the survey, a request to waive parental permission for teens under the age of 18 was approved by the Virginia Tech Institutional Review Board. Participants could take the online survey in English or Spanish one time either onsite with a researcher using an iPad tablet, or remotely at participants’ convenience; on average, participants took 15 min to complete the survey. Pre-screening questions at the beginning of the survey were used to filter out respondents that did not meet the eligibility criteria, who were prevented from consenting and proceeding to the full survey if they indicated that they did not meet the target audience criteria (e.g., excluded if not a mother between the ages of 21–36 years, teens and young adults younger/older than 14–20 years; residence outside of Fresno, CA or Hampton Roads, VA areas). Participants received a $10 gift card in-person or through email after completing the survey.

#### Survey measures

##### Awareness

To assess short-term awareness outcomes, the research team developed the following questions with input from the PHA and RWJF: (1) “Do you know what this brand or logo represents?” (featured in an image of the FNV logo along with the text of this question), (2) “Have you seen any versions of the FNV brand or logo around town or in your community?”, and (3) “Have you heard of the FNV Campaign?”; response categories were “yes”, “no”, or “unsure”. Respondents who selected “yes” to one or more of the three questions were coded as aware of the FNV Campaign, those who selected “no” or “unsure” were coded as unaware. These and other internally developed measures for this survey are available in the supplementary information as Additional file 1.

Three items that were used to assess short- to intermediate-term outcomes (i.e., fruit- and vegetable-related attitudes, beliefs, and encouragement) were adapted from the Food Attitudes and Behaviors Survey, which was developed based on a comprehensive literature review and expert content validity review of psychosocial constructs correlated with fruit and vegetable intake [38, 39]. Participants were asked to indicate their agreement with each statement on a five-point Likert scale ranging from “strongly disagree” to “strongly agree”, which were recoded for analysis to ordinal scale values 1 through 5, respectively.

##### Attitudes

Attitudes toward consuming new fruits and vegetables (i.e., neophobia) were assessed through agreement with the statement: “I enjoy trying new fruits and vegetables”.

##### Cognitive beliefs

Beliefs around perceived barriers to fruit and vegetable consumption were evaluated through respondents’ agreement with the statement “I just do not think of fruits and vegetables when I am looking for something to eat”; for the purposes of this evaluation, this statement was also used to indicate salience of fruits and vegetables.

##### Encouragement

Encouragement of others to eat fruits and vegetables was assessed through respondents’ agreement with the statement “I encourage my friends and family to eat fruits and vegetables”.

##### Behavioral intentions

Intentions related to buying and consuming fruits and vegetables were evaluated using two internally developed items that asked participants how likely they were to buy, and eat, “a fruit or vegetable over the next week”, as these were targeted intermediate outcomes of the FNV Campaign. The response categories for the two behavioral intention items were collapsed into unlikely (“unsure” or “unlikely”), and likely (“likely” or “very likely”) to dichotomize the responses to intending or not intending to buy and eat fruits and vegetables over the next week.

##### New fruit and vegetable intake

Intermediate-term behavioral outcomes related to consumption of new fruits and vegetables were assessed through an open-ended, internally developed question, which asked respondents to list any new fruits and/or vegetables they had tried; “What new fruits or vegetables have you tasted over the past 3 to 6 months that you have never eaten previously?” Written responses were transformed into a dichotomous response variable to indicate whether respondents reported trying any new fruits or vegetables.

##### Fruit and vegetable intake

Long-term outcomes related to daily fruit and vegetable intake frequency were assessed through items adapted from the validated Behavioral Risk Factor Surveillance System (BRFSS) fruit and vegetable screener [40]. Participants were asked through six items to indicate how many times they ate or drank 100% fruit juice, fruit, vegetable juice; and dark green, orange, and other vegetables during the past month, from “never” to “2 or more times per day”. At the time that this study was developed, legumes and beans were not widely promoted in the FNV Campaign and were not included in the vegetable intake assessment. Fruit and vegetable intake responses were converted into frequencies per day, which was summed into total daily fruit and vegetable intake frequency for analysis.

##### Demographic characteristics

The demographic characteristics assessed included sex, age, race/ethnicity, highest level of education, and geographic residence.

### Data analysis

All statistical analyses were performed separately for the mom and teen/young adult target audience respondents and by pilot market location using SPSS statistical software version 25 for Windows. Data analysis methods were selected and implemented with consultation from the Virginia Tech Statistical Applications and Innovations Group based on study objectives and data.

To assess awareness and fruit- and vegetable-related outcomes of respondents from the target audiences, descriptive statistics were calculated for awareness, fruit- and vegetable-related outcomes, and demographic characteristics. To examine whether awareness of the FNV Campaign was associated with differences in outcomes, all fruit- and vegetable-related outcomes (dependent variables) were compared between respondents categorized as aware and unaware (independent variable) in the following statistical tests: multivariate analysis of covariance (MANCOVA) was conducted to assess whether mean scores of fruit- and vegetable-related attitude, belief, and encouragement outcomes (dependent variables) differed between respondents aware of the FNV Campaign (independent variable). The use of MANCOVA to analyze several related variables is recommended over conducting separate univariate analyses to reduce the risk of family-wise error [41]. Associations between awareness and dichotomous variables for behavioral intentions and trying new fruits and vegetables were assessed using binary logistic regression. Analysis of covariance (ANCOVA) was used to determine whether mean daily fruit and vegetable intake frequency differed between aware and unaware respondents, to assess potential influence of the FNV Campaign on targeted intake behaviors.

Chi-squared tests were used to assess sample representativeness based on proportions of race/ethnicity and educational attainment as compared to proportions in the Fresno and Hampton Roads market locations [33, 34]. Race/ethnicity, sex (adolescents only), education (mothers only), age, and geographic location were included as covariates in the MANCOVA, ANCOVA, and logistic regression analyses because of potential biasing effects on outcomes related to fruit and vegetable consumption [42, 43].

## Results

After 2155 recorded responses were screened, 381 incomplete and ineligible responses (e.g., recorded age outside of criteria range) were removed, and the final sample contained 1604 eligible mom and teen/young adult complete survey responses across the two pilot locations. Table 1 shows the demographic characteristics of the respondents. The sample was made up of more target audience respondents who were mothers (53.6%; *n* = 860), and residents of the Hampton Roads, VA market (53.5%; *n* = 858). The proportions of racial and ethnic groups in the sample were generally representative of the pilot market racial and ethnic compositions. The majority of Fresno, CA respondents were Hispanic (53.4%) or White (20%), whereas most respondents from Hampton Roads, VA were White (42.7%) or Black (40.9%). Approximately 19% (*n* = 304) of the total sample and 41% of the teen/young adult sample was male. Representation of racial and ethnic groups in the sample did not significantly differ from the total population for the Fresno sample, but did for the Hampton Roads sample (*p* < 0.05), which had a higher proportion of Black respondents (42.7%) than in the Hampton Roads population [33, 34]. The percentage of the Fresno, CA or Hampton Roads, VA respondents with a high school education (or higher and a bachelor’s degree or higher did not significantly differ from the total population for either location [44].

**Table 1:** Demographic characteristics, FNV Campaign awareness, and fruit- and vegetable-related outcomes among teen and mom respondents, Fresno, California and Hampton Roads, Virginia, February – July 2017

	Total sample 1604 (100)	Teens/Young Adults 744 (46.4)	Moms 860 (53.6)
**Location, n (%)**			
Fresno, CA	746 (46.5)	392 (52.7)	354 (41.2)
Hampton Roads, VA	858 (53.5)	352 (47.3)	506 (58.8)
**Age, years, mean (SD)**	24.3 (7.0)	17.7 (2.0)	30.0 (4.1)
**Race/ethnicity, n (%)**			
Non-Hispanic White	515 (32.1)	247 (33.2)	268 (31.2)
Non-Hispanic Black	426 (26.6)	174 (23.4)	252 (29.3)
Hispanic	457 (28.5)	204 (27.4)	253 (29.4)
Other/multiracial	206 (12.8)	119 (16.0)	87 (10.1)
**Education, n (%)**			
Less than high school	374 (23.3)	308 (41.4)	66 (7.7)
High school graduate	323 (20.1)	190 (25.5)	133 (15.5)
Some college	562 (35.0)	240 (32.3)	322 (37.4)
College graduate or higher	345 (21.5)	6 (.8)	339 (39.4)
**Nutrition assistance program participation, n (%)**	483 (30.4)	155 (21.3)	328 (38.1)
**Aware of the FNV Campaign, n (%)**	315 (19.6)	167 (22.4)	148 (17.2)
**FV Attitudes, Beliefs, and Encouragement, mean (SD)**			
Attitude: I enjoy trying new FV^a^	4.14 (0.97)	3.92 (1.05)	4.33 (0.86)
Belief: I just do not think of FV when I am looking for something to eat^a,b^	3.94 (1.03)	3.71 (1.10)	3.13 (0.92)
Encouragement: I encourage my friends and family to eat FV^a^	4.06 (0.99)	3.74 (1.08)	4.33 (0.82)
**FV Behavioral Intentions, n (%)**			
Likely to buy FV over the next week^c^	1381 (86.1)	568 (76.3)	813 (94.5)
Likely to eat FV over the next week^c^	1491 (93.0)	665 (89.4)	826 (96.0)
**FV Behaviors**			
Tried a new fruit or vegetable in past 6 months, n (%)^d^	667 (41.6)	305 (41.0)	362 (42.10)
Daily FV intake frequency, mean (SD)^e^	3.17 (2.29)	2.75 (2.0)	3.53 (2.46)

*Abbreviations*: *FNV* Fruits & Veggies, *FV* Fruit and Vegetable, *SD* Standard Deviation

Data presented as number and percent of participants within each column subcategory for categorical variables and mean and standard deviation for continuous variables. Numbers may not equal total n because of unreported data and percentages may not add to 100 because of rounding

^a^Response options ranged from 1 (strongly disagree) to 5 (strongly agree)

^b^Item was reverse coded so that disagreement indicated lower perceived barrier to consumption

^c^Response options were collapsed into dichotomous variables, unlikely (“unsure” or “unlikely”), and likely (“likely” or “very likely”)

^d^Respondents were asked to list any new fruits or vegetables they had tried in the past 6 months. Written responses were transformed into a dichotomous response variable to indicate whether respondents reported trying any new fruits or vegetables

^e^Participants were asked to indicate how many times they consumed 6 categories of fruits and vegetables. Response options were converted to frequency per day: Never (0), 1 time/week (0.14), 2–3 times/week (0.36), 4–6 times/week (0.71), 1 time/day (1), 2 or more times/day (2), which was summed into total daily fruit and vegetable intake frequency for analysis

Approximately 20% (*n* = 315) of respondents reported that they were aware of the FNV Campaign, and a higher percentage of teens/young adults (22.4%) were aware than moms (17.2%). Table 2 summarizes the results comparing fruit and vegetable attitudes, beliefs, and encouragement between aware and unaware respondents, controlling for demographic characteristics. No statistically significant differences in attitudes, beliefs, and encouragement outcomes were found between the FNV aware versus unaware teen/young adult respondents. Among mothers, the only significant difference was that those aware of the FNV Campaign reported greater encouragement of fruit and vegetable intake than those who were unaware (*p* = 0.013). Outcomes by race/ethnicity, location, and sex (teens/young adults only) for the analyses reported in Tables 2, 3 and 4 are available in the online supplement.

**Table 2:** Fruit- and vegetable-related attitudes, beliefs, and encouragement by awareness of the FNV Campaign, Fresno, California and Hampton Roads, Virginia, February – July 2017

Survey Measure	Teens/Young Adults^b^		Moms^c^	
	Aware (mean ± SE)	Unaware (mean ± SE)	Aware (mean ± SE)	Unaware (mean ± SE)
**Attitude**: Enjoy trying new FV	4.02 ± 0.08	3.87 ± 0.04	4.48 ± 0.08	4.35 ± 0.04
**Belief**: Do not think of FV when looking for something to eat^a^	3.62 ± 0.09	3.69 ± 0.05	3.95 ± 0.08	4.01 ± 0.04
**Encouragement**: Encourage my friends and family to eat FV	3.82 ± 0.08	3.66 ± 0.05	4.50 ± 0.07	4.32 ± 0.04*

*Abbreviations*: *FNV* Fruits & Veggies, *FV* Fruits and Vegetables, *SE* Standard Error

Data for “Aware” and “Unaware” respondents presented as estimated marginal mean agreement ± SE; response options ranged from 1 (strongly disagree) to 5 (strongly agree)

^a^Item was reverse coded so that disagreement indicated lower perceived barrier to consumption

^b^MANCOVA with location, race/ethnicity, sex, and age as covariates

^c^MANCOVA with location, race/ethnicity, education, and age as covariates

* *p* < 0.05

**Table 3:** Fruit- and vegetable-related intentions and behavioral outcomes by awareness of the FNV Campaign, Fresno, California and Hampton Roads, Virginia, February – July 2017

Aware vs unaware odds of reporting:	Teens/Young Adults	Moms
	Odds Ratio^a^ (95% CI)	Odds Ratio^b^ (95% CI)
**Behavioral intentions**:		
Likely to buy FV over the next week	2.13 (1.30–3.50)**	1.08 (0.44–2.69)
Likely to eat FV over the next week	3.04 (1.32–7.0)**	3.17 (0.72–13.87)
**New fruit and vegetable intake**: Tried a new fruit or vegetable in the past 6 months	1.18 (0.83–1.69)	1.46 (1.02–2.09)*

*Abbreviations*: *FNV* Fruits & Veggies, *FV* Fruits and Vegetables, *CI* Confidence Interval

^a^Logistic regression with location, race/ethnicity, sex, and age as covariates

^b^Logistic regression with location, race/ethnicity, education, and age as covariates

* *p* < 0.05; **p < 0.01

**Table 4:** Estimated marginal means of daily fruit and vegetable intake frequency by FNV Campaign awareness, Fresno, California and Hampton Roads, Virginia, February – July 2017

	Teens/Young Adults^a^		Moms^b^	
	Aware (mean ± SE)	Unaware (mean ± SE)	Aware (mean ± SE)	Unaware (mean ± SE)
Daily FV intake frequency	2.82 ± 0.16	2.77 ± 0.09	3.73 ± 0.22	3.32 ± 0.12

*Abbreviations*: *FNV* Fruits & Veggies (FNV), *FV* Fruits and Vegetables, *SE* Standard Error

Data for “Aware” and “Unaware” respondents presented as estimated marginal mean frequency of fruit and vegetable intake per day ± SE

^a^ANCOVA with location, race/ethnicity, sex, and age as covariates

^b^ANCOVA with location, race/ethnicity, education, and age as covariates

Findings from the comparison of intention and behavioral outcomes between respondents categorized as aware and unaware of the FNV Campaign are shown in Table 3. Teens/young adults who were aware of the FNV Campaign, but not moms, significantly differed in their purchase and consumption intentions. Aware teens/young adults had 2.13 times higher odds of reporting the intention to buy (*p* = 0.003) and 3.04 times higher odds of reporting intention to eat (*p* = 0.009) fruits and vegetables than unaware teens/young adults. The odds of having tried a new fruit or vegetable in the past 6 months was 1.46 times higher for moms who were aware of the FNV Campaign (*p* = 0.04), compared to unaware moms.

Associations between awareness of the FNV Campaign and fruit and vegetable intake frequency are outlined in Table 4. There were no significant differences in mean daily fruit and vegetable intake frequency between aware and unaware mom and teen/young adult target audience respondents.

## Discussion

The present study sought to assess fruit- and vegetable-related outcomes among targeted audiences of the FNV Campaign in the Hampton Roads, VA and Fresno, CA markets where the FNV Campaign was first launched. It is the first study to empirically evaluate and report on fruit- and vegetable-related outcomes of the novel, industry-inspired FNV Campaign and had several notable findings.

Results from this evaluation indicated that the FNV Campaign IMC strategy reached targeted teen and mom audiences to raise brand awareness. Approximately 20% of target audience respondents reported awareness of the FNV Campaign 2 years after the launch in the pilot markets. This reported level of awareness is consistent with those reported for the *Fruits and Veggies–More Matters Campaign*; three years after the launch in 2007, awareness of the Campaign was 19% among target audience of moms aged 20–45 years [45]. Findings from formative and outcome evaluations of the adapted FNV Campaign for Wisconsin SNAP-Ed showed that awareness among millennial target audiences aged 18–34 years was 22% after only 6 months [19]. These findings suggest that the formative research to tailor the FNV Campaign intervention may have improved reach and relevance for local audiences and that partnership with SNAP-Ed may have enhanced evaluation and outcome reporting.

Our evaluation found positive associations between FNV Campaign awareness and some short- to intermediate-outcomes among target audience respondents. Results for mom target audience respondents suggest that awareness of the FNV Campaign was related to greater encouragement for others to eat fruits and vegetables. These improvements in intermediate outcomes are of importance as encouraging and modelling behaviors are important for establishing fruit and vegetable preferences and consumption among children [46, 47] and supporting greater intake among friends and family [48, 49]. However, no significant differences were found for encouragement among aware teen/young adult respondents, and there were no signuificant differences in fruit and vegetable attitude and belief outcomes in either teen/young adult or mom respondents.

Results indicated that awareness was associated with intentions to purchase and consume fruits and vegetables among teen/young adult respondents. This finding is especially relevant as adolescence is a time of increasing autonomy in making food choices among a variety of competing products [50], which may be an opportune time to increase intentions around fruit and vegetable consumption. Behavioral intentions may be a better target outcome among younger target audiences or those who are not the primary household shoppers, as the vast majority of moms respondents reported intentions to buy and eat fruits and vegetables (Table 1).

Positive behavioral outcomes were indicated for aware mom respondents, who reported trying a new fruit and vegetable significantly more often than unaware mothers, though it is unclear why this difference was not observed in teen/young adult respondents. This is an important finding as greater fruit and vegetable intake variety can also provide health benefits and reduce the risk of diet-related chronic diseases [51] in addition to increasing total consumption. Our evaluation did not find any changes in the target outcome of increased fruit and vegetable intake frequency among respondents who were aware of the FNV Campaign, though it is not surprising given that increasing consumption is regarded as a long-term goal (e.g., five or more years) for large-scale fruit and vegetable promotion campaigns [8]. However, positive produce sales results that were observed in Hampton Roads, VA Farm Fresh retail locations where the FNV Campaign was implemented suggest potential influence on behavior at the point-of-choice [19].

### Study limitations and strengths

The present study is subject to several limitations. First, the cross-sectional survey design cannot determine causality between the intervention and effects on outcomes. Additionally, our survey relied on self-reported data that are subject to selection, recall, and social desirability biases. Respondents who were aware of the FNV Campaign may have been more attuned to these promotions and already had more positive attitudes, beliefs, intentions, and behaviors related to fruits and vegetables. Additionally, the online survey format and non-probability, convenience sampling strategy may have excluded some populations with limited internet access and/or involvement with community organizations that supported recruitment efforts. Eligibility criteria were determined by the age groups that the FNV Campaign targeted and our survey did not assess maternity status among teen respondents, so our analysis of outcomes among moms may have overlooked younger mothers who were in the teen/young adult respondent category.

Additionally, this study was funded and initiated after the launch of the FNV Campaign and it has since been expanded nationally through national partnerships, and state- and local-level implementation through SNAP-Ed and food banks, while continuing efforts in the Fresno, CA and Hampton Roads, VA markets [23, 52–54]. By 2017, the PHA had engaged over 80 celebrity athletes and entertainers in FNV Campaign IMC promotions in the national markets [16]. As target audience characteristics were defined at the initiation of the grant process, results may not be generalizable to new locations and target demographic groups where the FNV Campaign has since scaled up and expanded. Findings from this study are strengthened by the large and racially and ethnically diverse sample of target audience participants, which was generally representative of the racial and ethnic demographic characteristics of the populations from which they were drawn.

The interpretation of study outcomes is limited without detailed documentation on the intermediate- and long-term target outcomes and criteria for success. Impressions, intensity, and duration of the FNV Campaign IMC strategies implemented over the two-year period prior to the survey implementation in 2017 have not been publicly reported and were beyond the scope of the present research. Future research is needed to elucidate potential relationships between the FNV Campaign content (e.g., messaging, celebrities, featured fruits and vegetables); dosage, duration, and medium of exposure; and changes in target audiences’ awareness and fruit- and vegetable-related outcomes.

Future evaluations of the FNV Campaign can contribute to food environment research by collecting and assessing the validity of objectively measured sales with self-reported measures of fruit and vegetable intake [55], including adequacy and diversity of intake, as measured in the 2015 Healthy Eating Index [56]. Rigorous process and outcome evaluations should be developed and initiated alongside FNV Campaign implementation to assess long-term progress. Evaluating the FNV Campaign in smaller-scale settings, such as schools and food retailers, could allow for more feasible intervention documentation and stronger evaluation designs (e.g., pre-post assessments, randomized control studies). To impact and sustain behavior change over the long-term, the FNV Campaign and other PSE initiatives should aim to improve fruit and vegetable access across multiple dimensions (i.e., availability, accessibility, affordability, acceptability, accommodation) while considering context-specific socio-ecological factors that guide and constrain health behaviors and health outcomes [55, 57, 58].

## Conclusions

This is the first independent evaluation to report fruit- and vegetable-related cognitive and behavioral outcomes for targeted populations in the pilot markets where the FNV Campaign was launched. This cross-sectional study found that about 20% of respondents reported awareness of the FNV Campaign brand after 2 years and that awareness was associated with limited but positive cognitive and behavioral outcomes among target audience respondents.

There is substantial potential to affect consumers’ consumption of fruits and vegetables through large-scale social marketing initiatives to meet both industry and public health goals and support widespread adoption. Further research is needed to understand how the FNV Campaign, and commercial marketing strategies broadly, can be utilized to increase fruit and vegetable consumption effectively and sustainably to recommended intake levels and improve health outcomes in diverse settings and populations. Future research should build upon these study findings to conduct longitudinal evaluations on consumption patterns in the two pilot markets and other locations where the FNV Campaign has expanded. Additionally, comparing cognitive and behavioral outcomes at similar points of Campaign duration could aid in identifying interventions, populations, or settings with greater potential to affect fruit and vegetable intake. Rigorous evaluations of the FNV Campaign are needed to build the evidence and leverage support for marketing campaigns to impact consumers’ food choice behaviors and health outcomes.

## Supplementary Information

**Additional file 1.** Survey Measures. Internally developed survey measures used to assess FNV Campaign awareness, intentions, and behaviors regarding fruit and vegetable consumption.

## Data Availability

The datasets used and/or analyzed during the current study are available from the corresponding author on reasonable request.

## Abbreviations

ANCOVA: Analysis of covariance
CA: California
FNV: Fruits & Veggies
IMC: Integrated marketing communication
MANCOVA: Multivariate Analysis of covariance
PHA: Partnership for a Healthier America
PSE: Policy, Systems and Environmental
SNAP-ED: Supplemental Nutrition Assistance Program-Education
US: United States
VA: Virginia
