# Supplementary material for: Awareness and outcomes of the fruits and veggies (FNV) campaign to promote fruit and vegetable consumption among targeted audiences in California and Virginia: a cross-sectional study
Source: BMC Public Health. 2021 Jun 9;21:1100. doi: 10.1186/s12889-021-11055-6 (PMC8191097; doi:10.1186/s12889-021-11055-6)
Supplement: Supplementary file 1 — Additional file 1. Survey Measures. Internally developed survey measures used to assess FNV Campaign awareness, intentions, and behaviors regarding fruit and vegetable consumption. [file 12889_2021_11055_MOESM1_ESM.docx]

**Additional file 1**. Survey Measures. Internally developed survey measures used to assess FNV Campaign awareness, intentions, and behaviors regarding fruit and vegetable consumption. Survey Measures

**Awareness**

Think about all of the advertising campaigns on television, the radio, the Internet and your mobile phone over the past 6 to 12 months.

Do you know what this brand or logo* represents?


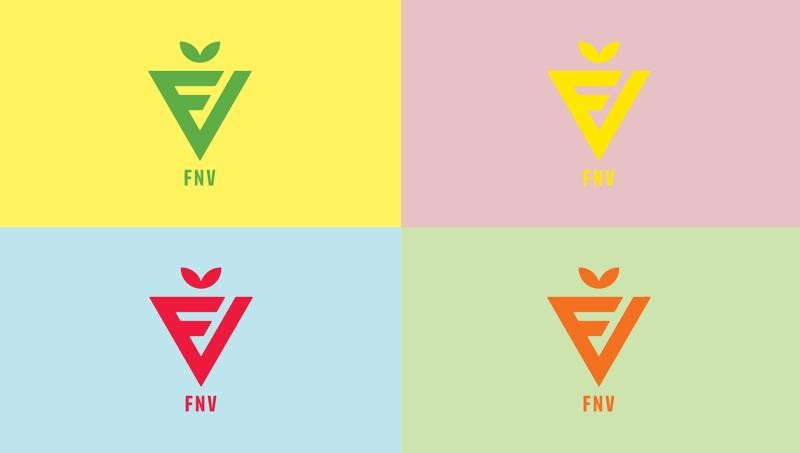
 *

Yes No Unsure

Have you seen any versions of the FNV brand or logo around town or in your community?

Yes No Unsure

Have you heard of the FNV Campaign?

Yes No Unsure

**Behavioral intentions**

How likely are you to **purchase** a fruit or vegetable over the next week?

Very Likely Likely Unlikely Unsure

How likely are you to **eat** a fruit or vegetable over the next week?

Very Likely Likely Unlikely Unsure

**New fruit and vegetable intake**

What new fruits or vegetables have you tasted over the past 3 to 6 months that you have never eaten previously?

*Sources: Partnership for a Healthier America. FNV Campaign. https://www.ahealthieramerica.org/fnv-fruits-vegetables-19 and http://noahswork.com/FNV.

The trademarked image used in the survey is intended for non-commercial educational purposes only. Their use is allowed for non-commercial purposes through the U.S. Nominative Fair Use Law that protect free speech over trademark infringement. More information about fair use is at https://www.copyright.gov/fair-use/more-info.html.
